# Supplementary material for: The Superior Laryngeal Nerve and Its Vulnerability in Surgeries of the Neck
Source: Diagnostics (Basel). 2021 Jul 12;11(7):1243. doi: 10.3390/diagnostics11071243 (PMC8305207; doi:10.3390/diagnostics11071243)
Supplement: Supplementary file 1 [file diagnostics-11-01243-s001.zip › diagnostics-1276154-SI.pdf]

Supplementary Table S1:

Study	Year	Major findings reported
Cheruiyot et al [1].	2018	The ebSLN coursed medial to the superior thyroid artery in 70.98% of all cases. Pre-operative planning and performance of operations in the neck is critical to reduce the incidences of iatrogenic EBSLN injury.
Chuang et al [9].	2010	In accord with the Cernea classification, the proportion of ebSLN variation are as follows: type 1 was 16.2%, type2a was 39.5%, and type 2 was 38.3%. Additionally, racial variations in the anatomy of the ebSLN exists between the white and Chinese population.
Dessie [10]	2018	The mean distance from the upper pole of the thyroid gland to the level where an external branch of superior laryngeal nerve turns medially from superior thyroid artery was found to be 1.04cm.
Estrela et al [11].	2011	There is a variation in the proximity relation between the ebSLN and the superior pole of the thyroid gland in which the distance may range from 3.25 to 15.75 mm
Guven et al [12].	2020	Of the total 29 ibSLN, 17 were divided into three branches and 12 into two branches. The ibSLN penetrated the thyrohyoid membrane at a mean distance of 12.0 ± 2.61 mm from the superior border of the thyroid cartilage and mean distance of 9.34 ± 1.65mm from the inferior border of the hyoid bone.
N'guessan et al [13].	2010	The situation of the ebSLN in relation to the branches of the superior thyroid artery were non-intermingled in 75% of cases and intermingled in 25% of the cases.
Sreedharan et al [14].	2018	The ebSLN ran ventrally and parallel to the STA, however, it is important to identify the branching point of the STA from the ECA or CCA. It is important to rule out anomalous origin of superior thyroid artery and verify its origin
Whitfield et al [15].	2010	The ebSLN entered the cricothyroid muscle at a median distance of 14 mm lateral from the laryngeal prominence and 8 mm inferiorly. The ebSLN was more likely to lie in an "at risk" location if the subject was less than 160 cm tall.
Yalcin et al [16].	2013	The topography of the ebSLN showed much variability in its relationship to the STA than is described in the literature, specifically in relation to the origin of the STA. Such variations should be kept in mind during surgery of the anterior neck.
